# Toxicity and population structure of the Rough‐Skinned Newt (*Taricha granulosa*) outside the range of an arms race with resistant predators

**DOI:** 10.1002/ece3.2068

**Published:** 2016-03-17

**Authors:** Michael T.J. Hague, Leleña A. Avila, Charles T. Hanifin, W. Andrew Snedden, Amber N. Stokes, Edmund D. Brodie, Edmund D. Brodie

**Affiliations:** ^1^ Department of BiologyUniversity of VirginiaCharlottesvilleVirginia; ^2^ Museum of Vertebrate ZoologyUniversity of CaliforniaBerkeleyCalifornia; ^3^ Department of BiologyUtah State University, Uintah Basin CampusVernalUtah; ^4^ VictoriaBCCanada; ^5^ Department of BiologyCalifornia State UniversityBakersfieldCalifornia; ^6^ Department of BiologyUtah State UniversityLoganUtah

**Keywords:** Arms race, coevolution, *Taricha granulosa*, tetrodotoxin

## Abstract

Species interactions, and their fitness consequences, vary across the geographic range of a coevolutionary relationship. This spatial heterogeneity in reciprocal selection is predicted to generate a geographic mosaic of local adaptation, wherein coevolutionary traits are phenotypically variable from one location to the next. Under this framework, allopatric populations should lack variation in coevolutionary traits due to the absence of reciprocal selection. We examine phenotypic variation in tetrodotoxin (TTX) toxicity of the Rough‐Skinned Newt (*Taricha granulosa*) in regions of allopatry with its TTX‐resistant predator, the Common Garter Snake (*Thamnophis sirtalis*). In sympatry, geographic patterns of phenotypic exaggeration in toxicity and toxin‐resistance are closely correlated in prey and predator, implying that reciprocal selection drives phenotypic variation in coevolutionary traits. Therefore, in allopatry with TTX‐resistant predators, we expect to find uniformly low levels of newt toxicity. We characterized TTX toxicity in northwestern North America, including the Alaskan panhandle where *Ta. granulosa* occur in allopatry with *Th. sirtalis*. First, we used microsatellite markers to estimate population genetic structure and determine if any phenotypic variation in toxicity might be explained by historical divergence. We found northern populations of *Ta. granulosa* generally lacked population structure in a pattern consistent with northern range expansion after the Pleistocene. Next, we chose a cluster of sites in Alaska, which uniformly lacked genetic divergence, to test for phenotypic divergence in toxicity. As predicted, overall levels of newt toxicity were low; however, we also detected unexpected among‐ and within‐population variation in toxicity. Most notably, a small number of individuals contained large doses of TTX that rival means of toxic populations in sympatry with *Th. sirtalis*. Phenotypic variation in toxicity, despite limited neutral genetic divergence, suggests that factors other than reciprocal selection with *Th. sirtalis* likely contribute to geographic patterns of toxicity in *Ta. granulosa*.

## Introduction

Coevolution occurs across a heterogeneous landscape of reciprocal selection, where species interactions and their fitness consequences vary from one location to the next (Thompson 2005). Reciprocal selection drives adaptive evolution at the phenotypic interface of coevolution – the set of traits that mediate the coevolutionary interaction (Brodie and Brodie 1999b; Brodie and Ridenhour 2003). Consequently, spatial variation in the form and intensity of reciprocal selection is predicted to generate a geographic mosaic of local adaptation to coevolutionary dynamics (Thompson 2005; e.g. Thompson 1997; Brodie et al. 2002; Nash et al. 2008). If among‐population phenotypic variation in coevolutionary traits is determined entirely by the heterogeneity of reciprocal selection, then allopatric populations should have limited phenotypic variation because of the absence of reciprocal selection. In antagonistic interactions, phenotypic exaggeration of traits like parasite virulence or prey toxicity should be uniformly limited in regions of allopatry with a natural enemy because exaggerated trait values are predicted to come at a physiological cost or trade‐off with other fitness components (Vermeij 1994; Abrams 2000; Rigby and Jokela 2000).

Here, we characterize variation in toxicity of the Rough‐Skinned Newt (*Taricha granulosa*) in allopatry with its toxin‐resistant predator, the Common Garter Snake (*Thamnophis sirtalis*), to test the hypothesis that phenotypic variation in a coevolutionary trait is limited in the absence of reciprocal selection with a natural enemy. *Taricha granulosa* and other congeners possess tetrodotoxin (TTX), a lethal neurotoxin that deters most predators. However, multiple species of garter snake, including *Th. sirtalis*, independently evolved resistance to the toxin (Geffeney et al. 2002, 2005; Feldman et al. 2009). Geographic patterns of phenotypic exaggeration in newt toxicity and snake TTX resistance are closely correlated across the co‐occurring range of the species in western North America, implying the existence of strong reciprocal selection (Hanifin et al. 2008). TTX resistance in western *Th. sirtalis* is clearly a derived trait (Motychak et al. 1999), and western populations in allopatry with *Taricha* generally lack exaggerated resistance (Brodie et al. 2002; Hanifin et al. 2008). However, the degree to which toxicity of *Taricha* varies in allopatry with *Th. sirtalis* is unknown.

**Figure 1:**
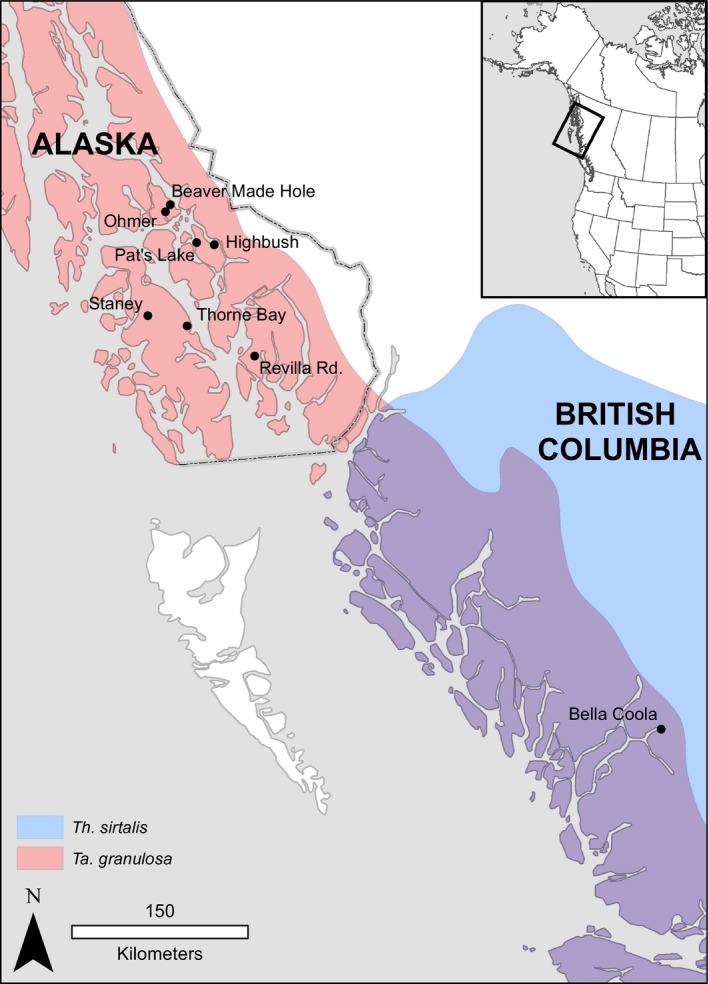
Sampling locations in southeastern Alaska and western British Columbia. The hypothesized geographic range is shown for *Th. sirtalis* (Rossman et al. 1996; Frost et al. 2015) and *Ta. granulosa* (Nussbaum and Brodie 1981; AmphibiaWeb 2016); however, the precise range boundaries of each species are not known.

We examine newt toxicity in northwestern North America, one of the few geographic regions where *Ta. granulosa* occur outside the range of any known TTX‐resistant predator, including *Th. sirtalis*. The range of *Ta. granulosa* extends north through the Alaskan panhandle (Nussbaum and Brodie 1981), whereas *Th. sirtalis* has been documented only as far north as central British Columbia (Fig. 1; Rossman et al. 1996). Despite anecdotal accounts, there are no photographs or voucher specimen of *Th. sirtalis* in Alaska (Neuman‐Lee et al. 2011). In this study, we were able to confirm the presence of *Th. sirtalis* as far north as the tip of Vancouver Island and Kitmat, BC, but we found no evidence of any natural populations of *Thamnophis* in Alaska.

First, we use neutral microsatellites to characterize population structure of *Ta. granulosa* in British Columbia and Alaska because biogeography provides an important context for which to understand geographic patterns of phenotypic variation (Thompson 2005; Knowles and Carstens 2007). For example, comparisons of toxicity among phylogenetically divergent populations would potentially be confounded by historical relationships. To avoid the confounding effects of phylogeny, we examine variation in toxicity among a geographic cluster of sites in Alaska where populations of *Ta. granulosa* lack neutral genetic subdivision.

We expect selection in the absence of a TTX‐resistant predator to favor reduced toxicity, particularly if TTX production comes at a physiological cost or trade‐off, as is predicted for increasing trait values in arms races (Vermeij 1994; Abrams 2000; Rigby and Jokela 2000). Alternatively, if the toxicity of *Ta. granulosa* varies significantly among Alaskan sites (despite a lack of neutral genetic divergence), factors other than reciprocal selection with *Th. sirtalis* likely contribute to variation in toxicity.

## Material and Methods

### Sampling

*Taricha granulosa* were sampled in April and May of 2004. We sampled from ponds in Bella Coola, British Columbia (within the range of *Th. sirtalis*) and on coastal islands of the Alaskan panhandle, including Revillagigedo, Wrangell, Mitkoff, and Prince of Wales islands (Table 1, Fig. 1). The following methods were conducted using an approved Institutional Animal Care and Use Committee protocol. We removed tail tip tissue from each newt for the genetic analysis and then used a human biopsy skin punch (Acuderm^®^ Inc., Ft. Lauderdale, FL, USA) to collect a 5 mm diameter dorsal skin punch for the TTX assay. In addition to the northern samples, we also collected genetic data from from a population of *Ta. granulosa* at Ledson Marsh in Sonoma County, California. These samples were used in the analysis of geographic population structure to provide a comparison of genetic variation with a population located in the southern portion of the range of *Ta. granulosa* (see Discussion).

**Table 1:** Sample locality details and microsatellite genetic diversity statistics for sites sampled in Alaska (*AK*), British Columbia (*BC*), and California (*CA*). The number of individuals collected (*N*) and successfully genotyped (*n*), mean observed heterozygosity (*H*_*O*_), expected heterozygosity (*H*_*E*_), number of alleles (*N*_*A*_), and allelic richness (A), along with standard deviations (SD) are reported

Locality (Island)	Code	Latitude	Longitude	*N*	Microsatellite diversity				
					*n*	*H*_*O*_ ± SD	*H*_*E*_ ± SD	*N*_*A*_ ± SD	A ± SD
AK									
Beaver Made Hole (Mitkof)	BMM	55.47	−131.61	16	15	0.34 ± 0.36	0.35 ± 0.25	3.17 ± 1.60	2.31 ± 0.97
Ohmer Creek (Mitkof)	OCM	56.33	−132.09	6	6	0.31 ± 0.35	0.29 ± 0.33	2.33 ± 1.37	2.25 ± 1.30
Pat's Lake (Wrangell)	PLW	56.35	−132.34	12	12	0.32 ± 0.30	0.32 ± 0.30	2.83 ± 1.47	2.27 ± 1.12
Highbush (Wrangell)	HBW	56.65	−132.68	19	16	0.29 ± 0.23	0.37 ± 0.27	2.67 ± 1.21	2.30 ± 0.95
Revilla Road (Revillagigedo)	RRR	56.60	−132.75	19	19	0.39 ± 0.22	0.42 ± 0.25	4.00 ± 1.55	2.71 ± 1.00
Staney Creek (Prince of Wales)	SCP	55.72	−132.51	16	16	0.37 ± 0.27	0.37 ± 0.25	3.50 ± 1.64	2.49 ± 1.08
Thorne Bay (Prince of Wales)	TBP	55.81	−133.04	20	20	0.32 ± 0.23	0.31 ± 0.21	3.00 ± 0.89	2.08 ± 0.65
BC									
Bella Coola	BCB	52.38	−126.58	32	32	0.53 ± 0.19	0.49 ± 0.16	3.50 ± 1.23	2.71 ± 0.74
CA									
Ledson Marsh	LMG	38.45	−122.65	20	20	0.65 ± 0.16	0.75 ± 0.13	7.60 ± 3.78	4.65 ± 1.61

### Genetic analysis

We extracted and purified genomic DNA from tail tissue using the DNeasy Blood & Tissue kit (Qiagen, Inc.). A total of six microsatellite loci – Tgr01, Tgr02, Tgr04, Tgr06, Tgr10, and Tgr14 – were amplified by adapting protocols from Jones et al. (2001). Microsatellites were run on a 3730xl 96‐Capillary Genetic Analyzer at the DNA Analysis Facility at Yale University and scored using GeneMarker v. 2.2.0 (SoftGenetics, State College, PA, USA). We were unable to amplify Tgr04 in the samples from Ledson Marsh, CA. We first estimated the frequency of null alleles for each locus using FREENA (Chapuis and Estoup 2007). GENEPOP (Raymond and Rousset 1995) was used to test for linkage disequilibrium between pairs of loci in each population using a log likelihood ratio test. The same program was used to test for departures from Hardy‐Weinberg equilibrium (HWE) for each locus and population. We adjusted *P*‐values with a sequential‐Bonferroni correction (Holm 1979). Next, we estimated population genetic diversity, including observed heterozygosity (*H*_O_), expected heterozygosity (*H*_*E*_), and number of alleles (*N*_*A*_) in ARLEQUIN 3.5.1.3 (Excoffier and Lischer 2010). We also used FSTAT (Goudet 1995) to estimate allelic richness (A) for each population.

To assess population structure, we used ARLEQUIN to estimate pairwise *F*_ST_ values between sampling sites. Statistical significance was obtained by permuting the samples 1000 times, and *P*‐values were adjusted with a sequential‐Bonferroni correction. We chose to use *F*_ST_, as opposed to *R*_ST_ because it tends to produce more accurate estimates of genetic subdivision when population structure is weak (as expected in Alaskan populations) and when the sample sizes and number of loci are limited (Gaggiotti et al. 1999; Balloux and Goudet 2002). We also used an analysis of molecular variance (AMOVA; Excoffier et al. 1992) implemented in ARLEQUIN, which assigns genetic variation to different geographic levels (within and among populations) to obtain global *F*_ST_ values. We estimated genetic subdivision among individuals grouped in two different manners: (1) by sampling location and (2) by the presence/absence of detectable TTX. We used the R package “adegenet” to test for the presence of isolation by distance (IBD) by plotting pairwise *F*_ST_/(1 ‐ *F*_ST_) against the logarithm of pairwise geographic distance (Rousset 1997). Significance was assessed with permutation‐based Mantel tests.

To further investigate population structure in northern populations, we used a Bayesian clustering analysis to estimate the optimal number of genetic clusters (*K*), implemented in STRUCTURE v. 2.3.4 (Pritchard et al. 2000; Falush et al. 2003). We used the sampling locations as prior information and assumed a model with population admixture and correlated allele frequencies (Falush et al. 2003). The analysis ran with 500,000 iterations as burn‐in and we collected data from the following 1,000,000 iterations of MCMC in 10 independent runs for values of *K* ranging from one to eight (eight being the total number of northern sampling locations). STRUCTURE HARVESTER (Earl and vonHoldt 2012) was used to detect the most probable number of clusters using the Evanno's method (Δ*K*; Evanno et al. 2005). Membership probabilities (*Q*‐values) of the 10 runs for each value of the most probable *K* (*K* = 2) were averaged using CLUMPP v. 1.1.2 (Jakobsson and Rosenberg 2007) and graphed using DISTRUCT v. 1.1 (Rosenberg 2004).

### Phenotypic analysis

Tetrodotoxin was extracted from the dorsal skin punches as described by Hanifin et al. (2002, 2004, 2008). For each skin punch, we quantified the amount of TTX in 20 *μ*L of extract using fluorometric high performance liquid chromatography (HPLC). We then estimated dorsal skin concentration of TTX (mg/cm^2^ of skin), along with whole animal toxicity (mg) based on each individual's total skin area. TTX is uniformly distributed throughout the dorsal skin and dorsal levels of TTX are strongly predictive of toxicity in other skin regions (Hanifin et al. 2004). For each geographic location, we quantified mean whole animal TTX (mg) as well as the proportion of newts with any detectable TTX from the HPLC analysis (>0.001 mg of TTX).

To test for among‐site differences in TTX toxicity, we used a nonparametric Kruskal‐Wallis test. We also used a multiple logistic regression to test for among‐site differences in the proportion of newts with any detectable TTX (i.e., presence vs. absence of TTX). We included sex in the model to account for potential differences in toxicity among males and females (Hanifin et al. 2002). We used the “glm” function in *R* (R Core Team 2014) with the proportion of toxic newts as the dependent variable, and population, sex, and the population*sex interaction as predictor variables. Significance of predictor variables was tested through comparisons with reduced models using likelihood ratio tests. We also generated distance matrices describing among‐site differences in mean toxicity (mg TTX/cm^2^) and the proportion of detectably toxic newts. We then used Mantel tests and Redundancy Analysis (RDA) to test for correlations between phenotypic divergence, neutral genetic divergence, and geographic distance, and then used permutation‐based methods to assess significance.

## Results

### Geographic population structure

We did not detect linkage disequilibrium for any pair of loci in any of the populations. We did find evidence for departures from HWE for locus Tgr10 in the Highbush populations, but we included Tgr10 in our analyses because this pattern was not consistent across all populations. These two populations also had null allele frequency estimates of 21% and 20% respectively, suggesting that deviations from HWE may be due to the presence of null alleles. The other five loci also showed infrequent evidence of null alleles, but these patterns were inconsistent across populations so we included all loci in our analyses. Genetic diversity statistics are summarized in Table 1. The California population of *Ta. granulosa* had the highest level of within‐population genetic diversity, whereas northern sites in British Columbia and Alaska all had low‐to‐moderate levels of diversity.

Measures of population differentiation generally revealed low levels of genetic subdivision among northern sites, particularly within Alaska. Nearly all the pairwise *F*_ST_ estimates among the geographically clustered Alaskan sites were not significantly different from zero (Table 2). Almost all Alaskan sites were significantly differentiated from the more geographically distant Bella Coola site in British Columbia. In the AMOVA including Alaska and British Columbia, the partition of among‐site variation was moderate and significant (*F*_ST_ = 0.0705, *P* = <0.001); however, *F*_ST_ was lower when British Columbia was excluded from the analysis (*F*_ST_ = 0.0185, *P* = 0.016). Even when Alaskan individuals were grouped by island (rather than by sampling location), *F*_ST_ was still low (*F*_ST_ = 0.024, and *P* < 0.001). Alternatively, when Alaska individuals were grouped according to their presence/absence of detectable TTX, we found a nonsignificant *F*_ST_ value (*F*_ST_ = −0.0011, *P* = 0.478). The test for IBD revealed a significant positive correlation between genetic differentiation and geographic distance (Mantel test: *r *=* *0.703, *P* = 0.022), but this relationship disappeared when Bella Coola was removed from the analysis (*r *=* *0.232, *P* = 0.175).

**Table 2:** Pairwise *F*_ST_ values from six microsatellite loci (or five in the California population, indicated in italics). Sampling sites are grouped by island. Significant values after a sequential‐Bonferroni correction are shown in bold

Island	Island	Mitkof		Wrangell		Revillagigedo	Prince of Wales		BCB	LMG
	Population	BMM	OCM	PLW	HBW	RRR	SCP	TBP		
Mitkof	BMM	–								
	OCM	−0.024	–							
Wrangell	PLW	0.029	0.052	–						
	HBW	0.037	0.026	−0.004	–					
Revillagigedo	RRR	0.040	0.004	−0.037	0.000	–				
Prince of Wales	SCP	**0.119**	**0.122**	0.015	0.006	0.036	–			
	TBP	0.041	−0.004	−0.032	−0.016	−0.013	0.004	–		
	BCB	**0.123**	0.062	**0.121**	**0.135**	**0.107**	**0.133**	**0.117**	–	
	LMG	*0.346*	*0.312*	*0.357*	*0.306*	*0.319*	*0.348*	*0.372*	*0.308*	–

**Figure 2:**
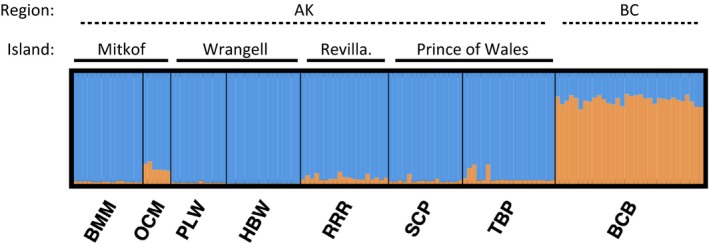
Bar plot obtained from STRUCTURE with *K *=* *2 for populations from Alaska (AK) and British Columbia (BC). Each vertical bar represents an individual and the height of each colored segment of a bar represents the probability of that individual's assignment to each cluster. Black vertical lines delineate sampling sites, which are labeled with codes from Table 1.

The STRUCTURE analysis revealed a similar pattern of population structure (Fig. 2). Both the Evanno's method (Δ*K*) and Ln*Pr*(X|*K*) supported a value of *K *=* *2. All Alaska individuals showed a high membership probability to one cluster, while British Columbia individuals predominantly grouped into another. To avoid overlooking fine scale population structure in Alaskan sites, we also ran a STRUCTURE analysis excluding individuals from British Columbia. The Ln*Pr*(X|*K*) values indicated *K *=* *1 as the most likely number of clusters and the bar plot of *K *=* *2 (not shown) identified all individuals across all populations as roughly equally admixed, indicating a lack of population structure.

### Phenotypic analysis

**Figure 3:**
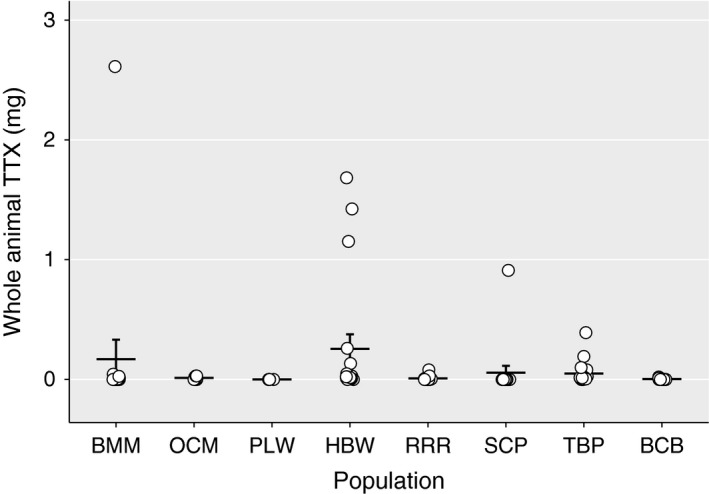
Among‐site variation in levels of whole animal TTX. Points are horizontally jittered. Black lines indicate site means (horizontal) ± standard error (vertical).

We detected significant among‐site differences in size‐adjusted estimates of skin TTX (Kruskal‐Wallis *χ* ^2^ = 44.62, *P* < 0.001, df = 7) and whole animal toxicity (Kruskal‐Wallis *χ* ^2^ = 40.40, *P* < 0.001, df = 7). The logistic regression also revealed a significant effect of collection site on the proportion of newts with detectable TTX (likelihood ratio *χ* ^2^ = 29.88, *P* < 0.001, df = 5). Sex and the sex*population interaction were not significant predictors in the model. We found large differences in the proportion of toxic newts across short geographic distances in Alaska, for example, the Pat's Lake site was devoid of newts with detectable levels of TTX, but only 15 km away at the Highbush site (on the same island), 94.7% of newts possessed detectable levels of TTX, with a mean toxicity of 0.2554 mg (Fig. 3). Several sites also had a small number of highly toxic individuals. One newt at Beaver Made Hole had an estimated total of 2.6 mg of TTX and three newts from Highbush had doses that exceeded 1 mg. For context, the lethal intraperitoneal dose of TTX required to kill a 20 g laboratory mouse in 30 min (i.e. a “mouse unit”) is roughly 0.2 *μ*g (Noguchi and Ebesu 2001). Thus, a newt with 2.6 mg of TTX contains enough toxin to kill approximately 13,000 mice.

The Mantel tests and RDA produced similar results, so here we only report Mantel test results (Table 3). We did not find a significant relationship between phenotypic divergence and neutral genetic divergence, which was unsurprising given the near complete lack of genetic divergence among Alaskan sites. The relationship between among‐site differences in the proportion of toxic newts and geographic distance was marginally significant in the Mantel test after a Bonferroni correction (*r *=* *−0.397, *P* = 0.0497), but not in the RDA. This pattern likely reflects the fact that the proportion of toxic newts at a given site can change drastically over short geographic distances.

**Table 3:** Results from Mantel tests for correlations among distance matrices from Alaskan sites. Significance was adjusted with a standard Bonferroni correction

Mantel tests	*r*	*P* value
*F*_ST_ + Mean TTX (mg/cm^2^)	−0.028	0.9059
*F*_ST_ + Proportion Toxic	−0.120	0.6039
Mean TTX (mg/cm^2^) + Geographic Distance	−0.227	0.3208
Proportion Toxic + Geographic Distance	−0.397	0.0497

## Discussion

Northern populations of *Ta. granulosa* generally lacked geographic population structure, particularly in Alaska. As predicted, overall population levels of newt toxicity were low outside the geographic range of TTX‐resistant predators. However, we found unexpected among‐ and within‐site variance in toxicity, which suggests that natural selection by resistant predators does not fully explain phenotypic variation in toxicity. First, we assess biogeographic structure in northern populations as context for interpreting geographic patterns of newt toxicity.

### Geographic population structure

Compared to California populations, *Ta. granulosa* in British Columbia and Alaska had reduced levels of genetic diversity. Estimates of pairwise *F*_ST_, the AMOVA, and the STRUCTURE analysis all suggest an overall lack of population structure in northern populations, particularly within Alaska. Jones et al. (2001) and Ridenhour et al. (2007) also reported low estimates of population subdivision among sites in Oregon and Washington, suggesting that *Ta. granulosa* may exhibit low site fidelity. We detected a genetic pattern consistent with IBD, but this reflects the fact that nearly all the Alaskan sites were significantly genetically differentiated from Bella Coola (Table 2, Fig. 2). Although newts from Bella Coola also had higher levels of heterozygosity than those from Alaska, these values were still low in comparison to the California sample.

The general lack of neutral genetic diversity in the northern samples is consistent with a northern postglacial range expansion after the Pleistocene (Hewitt and Ibrahim 2001). The low levels of genetic subdivision among island sites in Alaska, despite salt‐water barriers to dispersal, suggest the region was likely colonized recently by *Ta. granulosa*. Southeastern Alaska, Canada, and much of northwestern North America was either covered in ice or tundra‐like habitat during the Pleistocene (Barnosky et al. 1987; Josenhans et al. 1995; Mann and Hamilton 1995). Subsequently, *Ta. granulosa* and other codistributed taxa colonized northwestern North America in the last 10,000 years as the ice sheets retreated north (Kuchta and Tan 2005).

Elevated genetic diversity at the Bella Coola site relative to the Alaskan sites may result from a number of demographic processes. Populations of *Ta. granulosa* in Alaska sit near the northern limit of the species' range. A recent population bottleneck or extinction and recolonization event in Alaska could result in reduced population genetic variation compared to southern populations in Bella Coola. The Haida Gwaii region of coastal British Columbia (located proximate to Bella Coola) also has been proposed as a northern refugium for flora and fauna during the height of Pleistocene glaciation (Byun et al. 1997; Janzen et al. 2002; Shafer et al. 2010). A northern postglacial population expansion of *Ta. granulosa* out from the Haida Gwaii region could generate a pattern of lower genetic variation in Alaska compared to Bella Coola. A previous biogeographic analysis of allozymes and mitochondrial loci in *Ta. granulosa* suggested Alaskan populations were recently colonized as early as 10,000 years ago from Oregon or Washington (Kuchta and Tan 2005); however, the study lacked population sampling in British Columbia and could have missed cryptic refugia. Ultimately, more thorough sampling in British Columbia and Alaska is required to effectively discern among competing biogeographic hypotheses of postglacial expansion.

### Phenotypic analyses

As predicted, levels of newt toxicity among the genetically similar Alaskan sites were low. Mean toxicity values in Alaska were similar to those observed in populations that co‐occur with nonresistant populations of *Th. sirtalis* (Hanifin et al. 2008). The low levels of TTX observed in the majority of Alaskan samples may represent an evolutionary loss of toxicity. Brodie and Brodie (1991) suggested a similar loss of toxicity in *Ta. granulosa* on Vancouver Island, BC, where nontoxic newts were still found to be resistant to TTX. The TTX‐bearing phenotype appears to be ancestral in modern newts (family Salamandridae), a monophyletic group that includes *Ta. granulosa* (Hanifin and Gilly 2015). However, the evolutionary lability of TTX toxicity is unclear because genes associated with TTX biosynthesis have yet to be discovered and the newts may ultimately sequester the toxin through their diet or a bacterial symbiont (reviewed in Hanifin 2010). Although the ultimate source of TTX is unknown in *Ta. granulosa*, the apparent loss of toxicity in allopatry with a TTX‐resistant predator suggests that TTX synthesis or sequestration imposes a physiological cost. If TTX production requires a complicated biosynthetic pathway, selection may favor the loss of toxicity in the absence of a resistant predator (Williams 2010). For example, biosynthesis of a similar neurotoxin found in puffer fish, saxitoxin (STX), involves gene expression in a cluster of up to 26 genes (Moczydlowski 2013).

Despite low average levels of TTX toxicity throughout Alaska, we detected significant among‐site differences. The marginally significant inverse relationship between the proportion of toxic newts and geographic distance (Table 3) highlighted how differences in toxicity occur over short geographic distances, even on the same island (e.g., PLW and HBW on Wrangell Island). In addition, a small number of newts contained surprisingly large doses of TTX. These whole animal estimates of TTX (>1 mg per individual) rival the mean toxicity of populations of *Ta. granulosa* found in regions that co‐occur with TTX‐resistant *Th. sirtalis* (e.g. 1.628 mg of TTX in Tenmile, OR and 3.803 mg in McGribble, OR; Hanifin et al. 2008). The patterns of phenotypic variation in toxicity were incongruent with patterns of neutral microsatellite variation in Alaska, where populations were found to lack genetic subdivision. No evidence for a relationship between neutral genetic divergence and the presence/absence of TTX was apparent from the Mantel tests, RDA, or AMOVA.

The majority of sampled newts contained low or undetectable doses of TTX that are unlikely to be lethal to potential predators. Mean values of whole animal TTX in Alaskan populations were similar to those observed in newt populations in southern regions that co‐occur with nonresistant populations of *Th. sirtalis* (e.g. 0.011 mg in Skagit River, WA and 0.001 mg in Bear Ridge, CA; Hanifin et al. 2008). Although selection to deter TTX‐sensitive predators could conceivably maintain low levels of TTX in Alaskan newts, the large doses of TTX observed in a few individuals are extreme. *Thamnophis* species are the only known predators resistant enough to consume such large doses of TTX and there are no confirmed reports of sympatric garter snakes in Alaska. Consumption of *Taricha* by avian predators, including Western Grebes (*Aechmophorus occidentalis*; McAllister et al. 1997), a Mallard (*Anas platyrhynchos*; Storm 1948), domestic fowl (Pimentel 1952), and a Great Horned Owl (*Bubo virginianus*; Mobley and Stidham 2000) have resulted in death of the predators. Stokes et al. (2011) reported evisceration of *Taricha* by an unconfirmed avian predator at Ledson Marsh in California; however, local newts at the site have low levels of TTX. Successful consumption of a *Taricha* newt was reported for great blue herons (*Ardea herodias*; Fellers et al. 2008) and bullfrogs (*Rana catesbeiana*; Jennings and Cook 1998); however, both reports also came from regions where *Taricha* were nontoxic (Hanifin et al. 2008) and a separate study found that *Ta. granulosa* from toxic populations were lethal to both these predators (Brodie 1968). Small mammals may be another potential predator of *Ta. granulosa* in Alaska, but previous tests found ten genera of mammals to be highly sensitive to TTX, including rats (*Rattus rattus*), a stoat (*Mustela erminea*), a muskrat (*Ondatra zibethicus*), and a bobcat (*Lynx rufus*) (Brodie 1968). Adult newt toxicity may reflect selection for defense against predators at an earlier life stage. Egg toxicity of *Ta. granulosa* is positively correlated with maternal toxicity (Hanifin et al. 2003; Gall et al. 2012a). Moreover, caddisfly larvae (*Limnephilus flavastellus*) in Oregon are capable of preying on the eggs of *Ta. granulosa*, and appear to harbor some degree of resistance to TTX (Gall et al. 2011, 2012b).

The variance in toxicity of *Ta. granulosa* in allopatry with *Th. sirtalis* suggests that factors other than the coevolutionary interaction with resistant predators contribute to geographic patterns of phenotypic variation in toxicity. External factors, such as abiotic conditions, may affect the ability of newts to sequester or synthesize TTX. For example, marine taxa are generally thought to obtain TTX through the food chain or a bacterial symbiont, and high individual and regional variation in toxicity has been cited as evidence of an exogenous source of TTX in puffer fish (reviewed in Noguchi and Arakawa 2008). However, the source of TTX in newts is more controversial (Hanifin et al. 2008). When fed a nontoxic diet, captive *Ta. granulosa* can maintain and regenerate levels of TTX for extended periods (Hanifin et al. 2002; Cardall et al. 2004), but analogous tests in the fire‐bellied newt (*Cynops pyrrhogaster*) and the red‐spotted newt (*Notophthalmus viridescens*) appeared to result in a loss of toxicity (Yotsu‐Yamashita et al. 2012; Kudo et al. 2015).

Variation in toxicity in Alaska could also result from increased variance in TTX synthesis genes and genetic drift in the absence of selection for exaggerated toxicity. This alternative may be less likely, given the lack of neutral genetic variation and subdivision in Alaskan sites. The incongruence between phenotypic variation and the lack of neutral microsatellite variation suggests that among‐site variance in toxicity is not solely due to neutral drift. Admittedly, we cannot rule out the importance of genetic drift because the genetic basis of TTX synethesis is unknown. Presumably TTX toxicity in *Ta. granulosa* has some heritable genetic component that is susceptible to drift because southern populations have apparently evolved extreme toxicity in response to escalatory reciprocal selection with *Th. sirtalis* (Hanifin et al. 2008).

## Conclusion

As predicted, levels of toxicity were generally low in populations of *Ta. granulosa* in allopatry with TTX‐resistant predators. However, we also found evidence for among‐ and within‐population variation in toxicity, a pattern that appears to be inconsistent with neutral genetic population structure. The limited number of samples and microsatellite markers in this study may restrict our power to detect microgeographic population structure in Alaska, thus our results should be interpreted with caution. Nevertheless, estimates of *F*_ST_, tests for IBD, and the STRUCTURE analysis all suggest an overall pattern of limited population stucture among the geographic cluster of sites in Alaska, which decreases the likelihood that our population comparisons of TTX toxicity are confounded by deep phylogenetic divergence.

Characterizing toxicity of *Ta. granulosa* in allopatry with *Th. sirtalis* represents a critical step in inferrering how exogenous forces might influence coevolution in sympatry. These data emphasize that reciprocal selection is likely not the sole determinant of geographic patterns of toxicity in *Ta. granulosa*. Focusing only on phenotypic variation in sympatric populations would otherwise lead to a myopic interpretation of coevolutionary dynamics. The coevolutionary process occurs across space and time, which inevitability span variable ecologial and abiotic conditions. Factors unrelated to the interaction, like environmental conditions (Johnson et al. 2007; Williams 2010), physiological trade‐offs (Brodie and Brodie 1999a; Rigby and Jokela 2000), or selection from interactions with other organisms (Zangerl and Berenbaum 2003; Siepielski and Benkman 2004) can alter the evolutionary trajectory of coevolutionary traits. The contribution of exogenous factors should not be overlooked in the context of geographic patterns of adaptation at the phenotypic interface of coevolution.

## Conflict of Interest

None declared.
